# Extensive outbreak of colistin resistant, carbapenemase (*bla*_OXA-48_, *bla*_NDM_) producing *Klebsiella pneumoniae* in a large tertiary care hospital, India

**DOI:** 10.1186/s13756-021-01048-w

**Published:** 2022-01-06

**Authors:** Swati Sharma, Tuhina Banerjee, Ashok Kumar, Ghanshyam Yadav, Sriparna Basu

**Affiliations:** 1 Department of Microbiology, Institute of Medical Sciences, Banaras Hindu University, Varanasi, 221005 India; 2 Department of Pediatrics, Institute of Medical Sciences, Banaras Hindu University, Varanasi, India; 3 Department of Anaesthesiology, Institute of Medical Sciences, Banaras Hindu University, Varanasi, India; 4 Department of Neonatology, All India Institute of Medical Sciences, Rishikesh, India

**Keywords:** *bla*_OXA-48_, CRKp, Fatal, Hospital environment, ST5235, ST5313, Surveillance

## Abstract

**Background:**

Extensive drug resistance in *Klebsiella pneumoniae* (*K. pneumoniae*) causing major outbreaks in large hospitals is an emerging challenge. We describe a near fatal outbreak of colistin resistant, carbapenem resistant *K. pneumoniae* (CRKp) producing metallo beta-lactamases (*bla*_NDM_) and *bla*_OXA-48_ in the neonatal intensive care unit (NICU) at the background of a larger outbreak involving multiple parts of the hospital and the challenges in its containment.

**Methods:**

Following identification of an outbreak due to colistin resistant CRKp between April to June 2017 in the NICU, a thorough surveillance of similar cases and the hospital environment was performed to trace the source. All the isolated *K. pneumoniae* were tested for susceptibility to standard antibiotics by disc diffusion and microbroth dilution methods. Molecular detection of extended spectrum beta lactamases (ESBLs) and carbapenemases (classes A, B, D) genes was done. Enterobacterial repetitive intergenic consensus (ERIC) PCR and multi-locus sequence typing (MLST) was done to determine the genetic relatedness of the isolates. Characteristics of different sequence types were statistically compared (Student’s t-test).

**Results:**

A total of 45 K*. pneumoniae* isolates were studied from NICU (14 cases of neonatal sepsis), ICU (18 cases), other wards (7 cases) along with 6 isolates from hospital environment and human colonizers. The primary case was identified in the ICU. All the *K. pneumoniae* from NICU and 94.4% from the ICU were colistin resistant CRKp. Majority (59.37% and 56.25%) harbored *bla*_SHV_/*bla*_CTXM_ and *bla*_OXA-48_ genes, respectively. Two distinct sequence types ST5235 and ST5313 were noted with colistin resistance, distribution within the NICU and mortality as significant attributes of ST5235 (*p* < 0.05). The outbreak was contained with strengthening of the infection control practices and unintended short duration closure of the hospital.

**Conclusion:**

Large hospital outbreaks with considerable mortality can be caused by non-dominant clones of colistin resistant CRKp harboring *bla*_OXA-48_ and *bla*_NDM_ carbapenemases in endemic regions. The exact global impact of these sequence types should be further studied to prevent future fatal outbreaks.

## Introduction

In developing countries of Asia and Africa, *Klebsiella pneumoniae* (*K. pneumoniae*) has been the predominant pathogen responsible for nearly 50% of neonatal sepsis due to Gram negative organisms [1]. In addition, this organism also possesses the highest rates of antimicrobial resistance (AMR). Unfortunately, studies have reported alarmingly high resistance rates of nearly 83.35% among extensively drug resistant (XDR) *K. pneumoniae* in neonatal sepsis [2]. The rapid emergence and widespread dissemination of carbapenem resistance in *K. pneumoniae* has posed several global challenges especially with management of such infections. As against in Western countries, where carbapenem resistance is mediated by *K. pneumoniae* carbapenemase (KPC), in Asian countries including India, metallo beta-lactamases (MBLs) and Class D carbapenemases are the commonest causes [3]. Consequently, the scope of alternate therapy becomes still narrower. While newer antibiotic combinations can work for KPC producing *K. pneumoniae*, they are mostly ineffective against the MBLs and Class D carbapenemases.

Although genetic determinants of AMR are widely distributed in *K. pneumoniae*, the globally circulating clones of multidrug resistant (MDR) *K. pneumoniae* associated with major hospital outbreaks are seen to be common [4]. There is lack of data on the other circulating clones from regions with different epidemiological conditions or for those that acquire AMR under specific environmental challenges within the hospital. It has been seen that *K. pneumoniae* is present in the hospital environment more frequently than other coliforms [5]. It is very difficult to eradicate such environmental reservoirs due to adaptability and several interactions of *K. pneumoniae* with environmental stress factors. We had experienced a sustained outbreak of extended spectrum beta-lactamases (ESBLs) producing *K. pneumoniae* in the neonatal intensive care unit (NICU) in the past following which changes in antimicrobial stewardship were initiated and major outbreaks were contained [6]. In the present study, we describe a fatal outbreak of extensively drug resistant *K. pneumoniae* (XDRKp) in the NICU at the background of a larger outbreak involving the other parts of the hospital and the course of its containment.

## Materials and methods

### Study setting

This study was conducted in the Department of Microbiology and the associated tertiary care hospital, Varanasi, India. This 2000 bedded teaching hospital is the largest referral centre in the eastern part of the largest state of the country, catering to the needs of approximately 20 crore population in the adjoining catchment area. The study was carried out in between January 2017 to December 2020.

### Source of the study isolates

Between April to June 2017, the NICU of the tertiary care hospital was affected by an outbreak of *K. pneumoniae* with considerable mortality. As a part of infection control protocol, thorough environmental surveillance of the NICU and the labour room was performed. Further, to trace the source of the outbreak, *K. pneumoniae* infections were studied from the entire hospital during the same time. Microbiological surveillance of the hospital environment including hands of the healthcare workers was done for the entire hospital. Isolates of *K. pneumoniae* collected from relevant samples like blood culture for sepsis, pus for soft tissue infections, sputum/endotracheal aspirates for pneumonia by routine procedure was stored at -20^0^C, and further used for the study. Surveillance cultures from various parts of the hospital like bedrails, beddings, saline stands, monitors, wash basins and relevant objects in the immediate vicinity of the patients were collected from ICUs, wards and operation theatres (OTs). Culture surveillance was also done from all the neonates affected in the outbreak as well as those not affected by collection of anal, skin and umbilical swabs. Isolation and identification of *K. pneumoniae* was done by routine biochemical tests [7]. Confirmation of all the isolates was done in Vitek® 2 compact system (bioMérieux India Private Limited, India) and BD Phoenix™ M50 (Becton, Dickinson and company Diagnostics, India).

### Antimicrobial susceptibility testing

Susceptibility testing against the following antibiotics was done by Kirby Bauer modified disc diffusion method following the standard guidelines. Following discs were used: ampicillin/sulbactam (10/10 µg), piperacillin/tazobactam (100/10 µg), ceftazidime (30 µg), cefepime (30 µg), ceftriaxone (30 µg), imipenem (10 µg), meropenem (10 µg), gentamicin (10 µg), amikacin (30 µg), ciprofloxacin (5 µg) and levofloxacin (5 µg) (HiMedia Laboratories Pvt. Ltd, India) [8]. Further, minimum inhibitory concentration (MIC) against imipenem, meropenem and colistin (Sigma Aldrich Chemicals Pvt. Ltd, India) was determined by microdilution broth method [8]. *Escherichia coli* ATCC 25922 was used as quality control.

Further, detection of resistance determinants including ESBL genes (*bla*_TEM_, *bla*_SHV_, *bla*_CTXM -1,2,9)_ and class A/B/D carbapenemase encoding genes (*bla*_SME_, *bla*_NMC_, *bla*_GES_, *bla*_KPC_, *bla*_IMP_, *bla*_VIM_, *bla*_NDM_ and *bla*_OXA-48_) was done by multiplex-polymerase chain reaction (PCR) as described earlier [6, 9, 10]. Genomic DNA was extracted from pure isolated colonies of *K. pneumoniae* using QIAamp® DNA mini kit (Qiagen India Pvt. Ltd, India) as per manufacturer instruction. Completely characterized controls from previous publications were used as positive controls while molecular grade water was considered as negative control.

### Strain typing

Clonal diversity of the isolates was determined by enterobacterial repetitive intergenic consensus (ERIC) PCR. Amplification was done using the universal primers and under the reaction conditions as described elsewhere [11]. The amplified product was run on agarose gel electrophoresis and the bands were visualized by gel documentation (BioRad Laboratories India Pvt. Ltd, India). A dendrogram was drawn based on obtained band pattern by using NTSYS-pc version 2.0 software. Isolates with a similar ERIC pattern were considered as one ERIC type and isolates with inconsistent bands were classified into different ERIC types.

### Multilocus sequence typing (MLST)

MLST was done for all the isolates from NICU and for one representative isolate from each of the 100% similar ERIC cluster. Amplification of the seven-housekeeping genes *rpoB, gapA, mdh, pgi, PhoE infB* and *tonB* was done using the primer pairs and conditions as described previously [12]. The amplified PCR products were sent for Sanger sequencing (EzeDiagnon Healthcare Pvt. Ltd, India). Each forward and reverse sequences obtained were assembled by BioEdit v7.2.5 software. The assembled sequences were submitted to *Klebsiella pneumoniae* MLST database at the Pasteur Institute https://bigsdb.pasteur.fr/klebsiella/klebsiella.html for allele and sequence type determination.

### Statistical analysis

The characteristics of the isolates belonging to different STs were compared using independent ‘t’ test (MedCalc statistical software version 20.009).

## Results

### Outbreak description

The primary case of neonatal sepsis in the NICU was identified on 17^th^ April, following which up to June 2017, sudden surge of 14 cases of neonatal sepsis due to *K. pneumoniae* was noticed with nearly 100% mortality (12/14, 85.71%). All the *K. pneumoniae* isolates belonged to ST5235. These affected neonates were delivered in the same hospital and had history of prematurity, preterm, respiratory distress, low birth weight and were therefore transferred to the NICU. The ongoing antibiotic policy in the NICU was to administer piperacillin-tazobactam and amikacin empirically followed by culture-based treatment. However, definitive treatment for these XDR isolates could not be reached even with several combinations of imipenem/meropenem, amikacin and vancomycin.

**Fig. 1:**
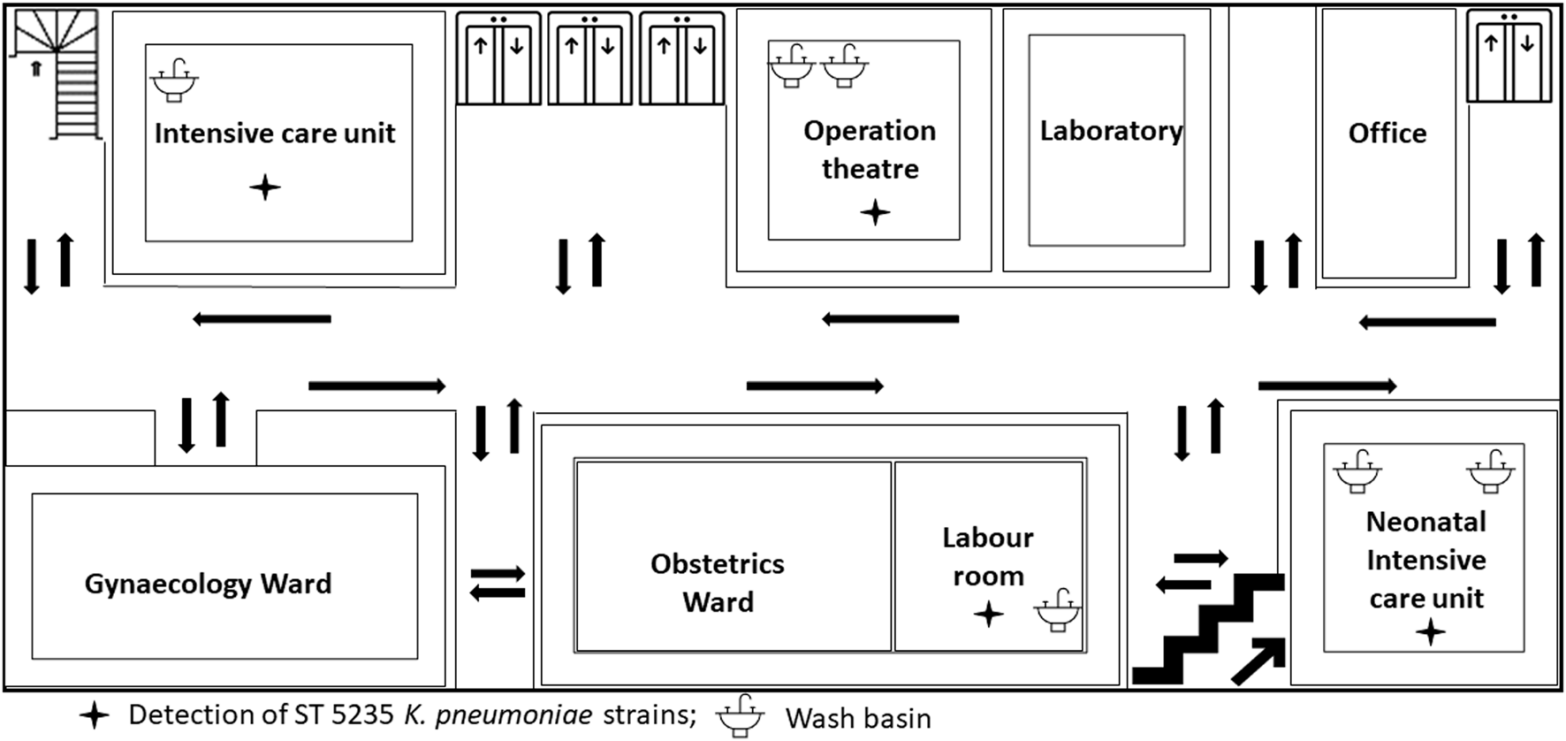
The floor plan (1st floor) of the hospital showing the location of the NICU and ICU

During the outbreak, environmental surveillance of NICU and labour room revealed the presence of *K. pneumoniae* in the handwash in-use in the NICU (ST5313) and wash basin of the labour room (ST5235). Besides, *K. pneumoniae* was also isolated from wash basins of Emergency OTs (ST5235) located in immediate vicinity of NICU during this period. The floor plan of the NICU and the other related wards and OTs has been shown in Fig. 1. Culture surveillance from the admitted neonates showed the presence of *K. pneumoniae* in umbilical/skin swabs of 3 neonates with *K. pneumoniae* sepsis. No anal colonization with *K. pneumoniae* was seen in any of the neonates. The affected neonates were transferred from the hospital’s labour room to the NICU through staff from both the facilities. The beddings of the newborn were carried from labour room to the NICU without any cleaning or disinfection procedure. The duties of the nursing staffs were rotational in nature with extensive exchange in between the wards and the ICUs.

**Fig. 2:**
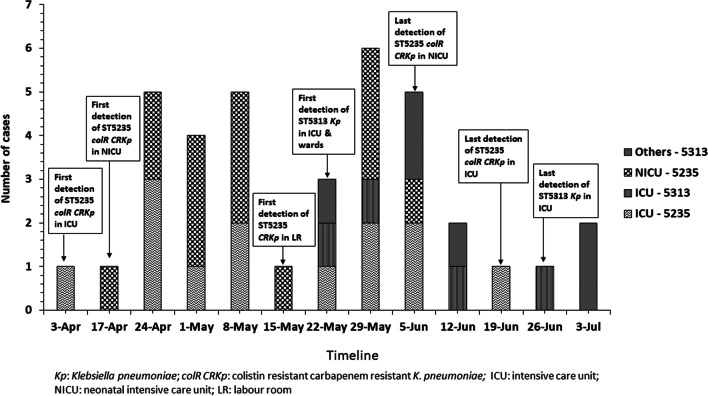
Distribution of the cases and their locations during the outbreak period (April-July 2017)

In relevance to the outbreak in NICU, it was revealed that the primary case of *K. pneumoniae* infection (ST5235) initiated in the adult ICU on 3rd April, 2017. Following this, 18 cases of infections due to *K. pneumoniae* was noted in the ICU till July 2017. The isolates were recovered from various infections like nosocomial pneumonia including those associated with ventilators (15) and sepsis (3). Of these, isolates from both ST5235 and ST5313 were detected. Further, during this period, 7 more isolates of *K. pneumoniae* from different wards like 3 cases of surgical site infections (SSIs) in male surgical ward (MSW) and 1 each from chest (pneumonia), urology (catheter associated urinary tract infection), dermatology (SSI) and radiotherapy (sepsis) wards were identified. The distribution of the cases throughout the outbreak period has been shown in Fig. 2.

### Outbreak containment

**Fig. 3:**
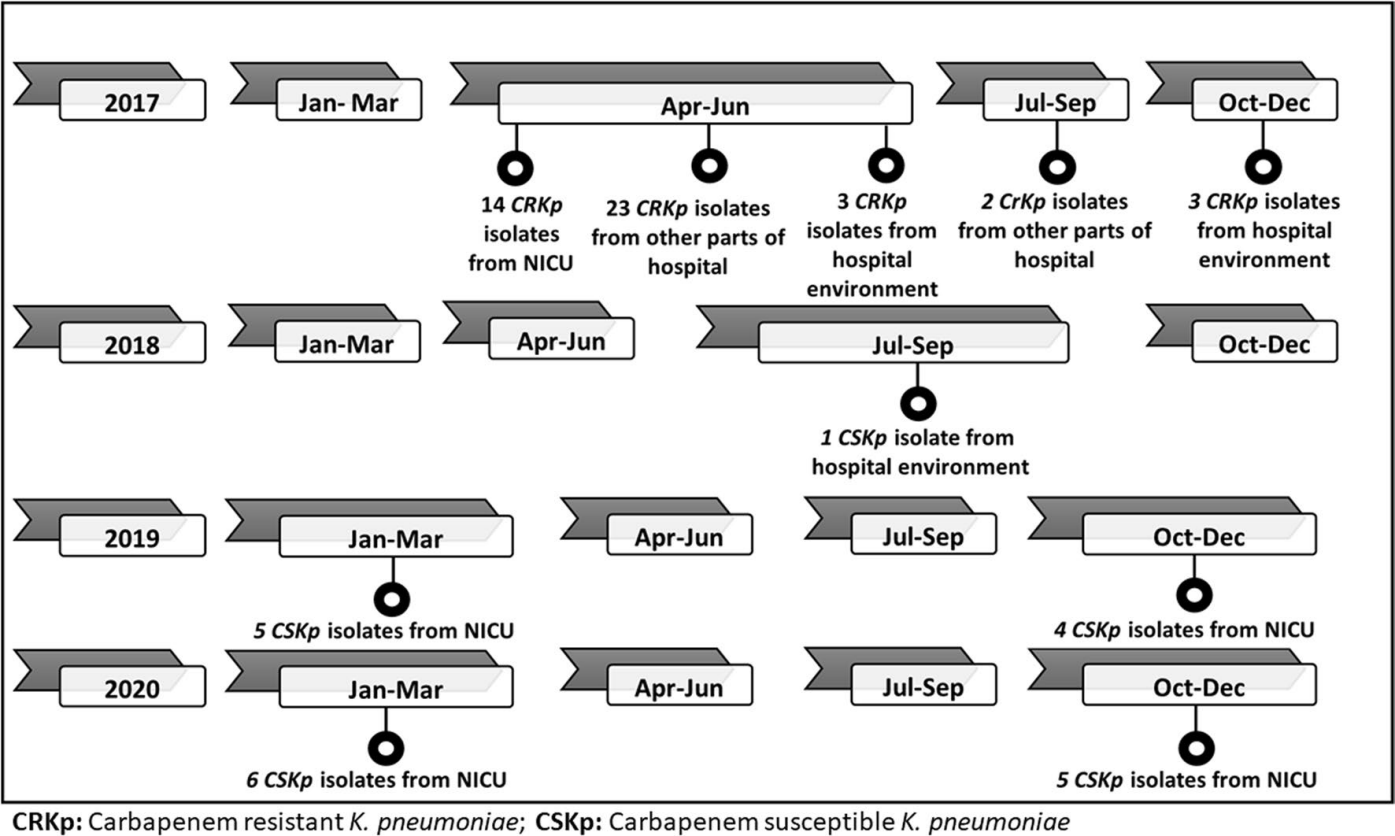
Isolation of *K. pneumoniae* from various sources in the study period (2017–2020)

Multidirectional infection control (IC) approaches were strengthened despite the fact that the NICU maintained adequate IC practices. It was observed that in the NICU, reprocessing of ventilation equipments was usually avoided. However due to limitation in resources, ventilation circuits were re-used after surface disinfection with 1% hypochlorite in the NICU itself. Similarly, re-use of these circuits was more common in the adult ICU and other wards. However, cleaning of these equipment prior to disinfection was often compromised due to excessive patient turnover. All the affected neonates were cohorted from the unaffected ones. Specific interventions like thorough surface disinfection at least twice daily, hand hygiene monitoring, staff education was prioritized. Standard operating procedures (SOPs) on preparation and choice of surface disinfection was introduced. Surface cleaning of bed rails and associated items were irregularly and improperly done with 0.1% bleaching powder, which was often offensive for use by the staff. Quaternary ammonium compounds were introduced as surface disinfectants. Number of unnecessary foot falls and movement of healthcare workers inside the NICU were restricted. Due to excessive patient turnover, there was no scope for closure of the neonatal unit. However, as the outbreak continued, there was sudden unprecedented suspension of all admissions throughout the hospital due to administrative issues relating to faulty oxygen cylinders for 3 days (9th–11th June, 2017). Following closure, the number of existing and new admissions of patients in the hospital were gradually reduced. Surprisingly, the isolation of *K. pneumoniae* with similar profile slowed down with no more cases from the NICU since 6^th^ June 2017 and in the ICU since 26th June 2017. The last isolate of *K. pneumoniae* of ST5235 was obtained on 6^th^ July from cases in Dermatology and Radiotherapy wards. Thereafter, the NICU was continuously monitored till December 2020. Meanwhile specific interventions like major infrastructural changes with separate facilities for in-born and out-born neonates in the NICU, recruitment of dedicated nursing staff for the NICU, rigorous implementation and monitoring of IC practices, strengthening of the antimicrobial stewardship programme and regular gut culture surveillance and microbiological surveillance of the NICU environment were initiated. During this period, on few occasions, *K. pneumoniae* was isolated from the environment at different parts of the hospital. However, cases of neonatal sepsis due to *K. pneumoniae* with susceptible profile were dispersed throughout the year and no major clustering of isolates with similar susceptibility profile was seen. Gut colonization with *K. pneumoniae* was not detected in the neonates throughout the study period. The isolation of *K. pneumoniae* from the various sources during the entire study period has been shown in Fig. 3.

### Antimicrobial susceptibility profile of the isolates

All the 14 isolates of *K. pneumoniae* from neonatal sepsis were resistant to all the tested antibiotics including carbapenems and colistin. Among the isolates from patients in other parts of the hospital, 17 of the 18 isolates from 18 cases of sepsis (3) and pneumonia (15) in the ICU were also resistant to all the tested antibiotics including carbapenems and colistin. Additionally, the remaining 7 isolates from other wards were susceptible to carbapenems and colistin with the exception of 1 isolate from MSW which was resistant to carbapenem.

Among the total 32 CRKp isolates from ICU and the NICU, different ESBL genes were present with a combination of both *bla*_SHV_ and *bla*_CTXM_ being commonest in majority of the isolates (19/32, 59.37%). All the CRKp isolates harboured class B carbapenemases with majority (18/32, 56.25%) showing the presence of *bla*_OXA-48_ and 21.87% (7) *bla*_NDM_ genes.

### Clonal typing

**Fig. 4:**
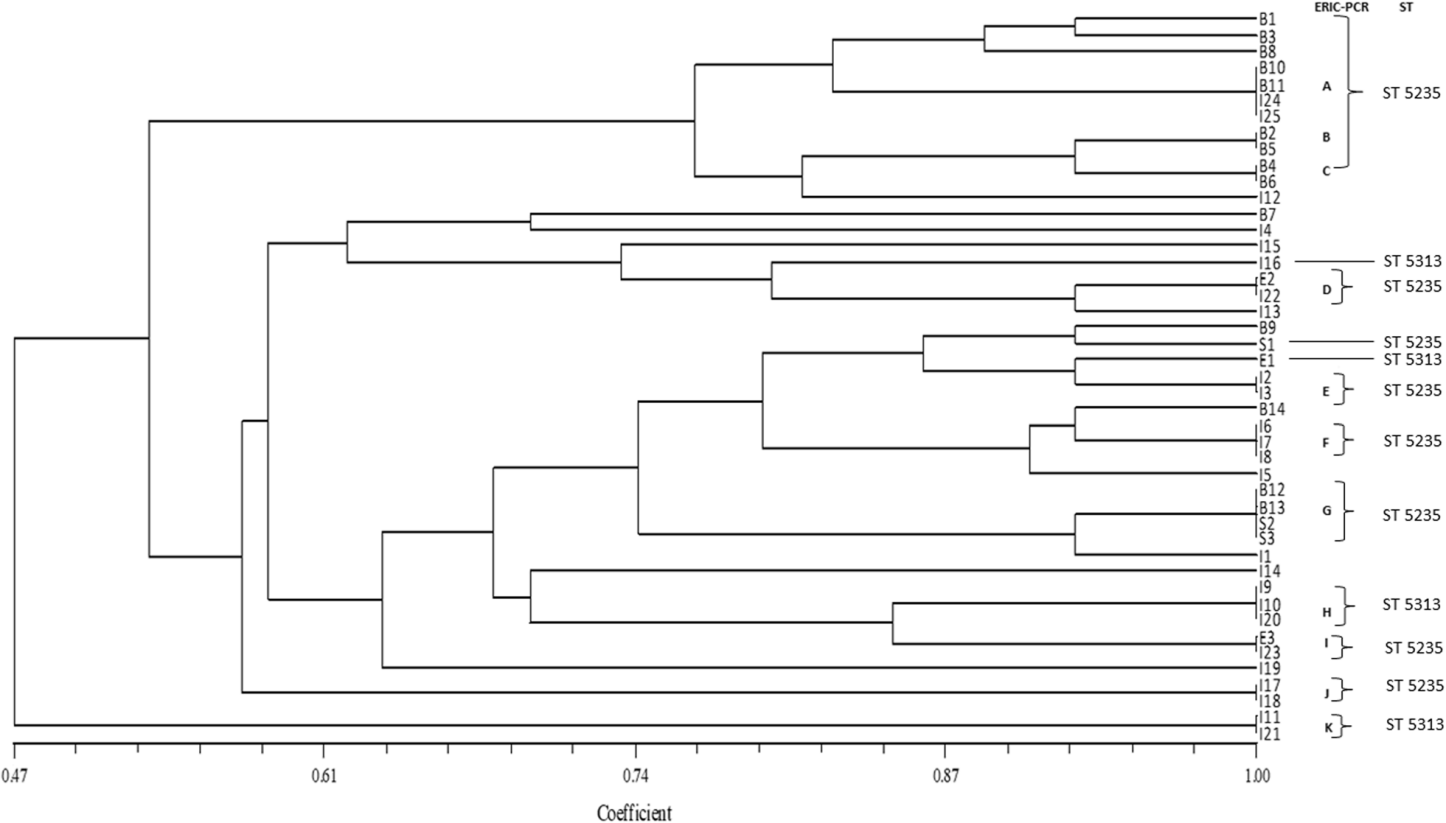
Clonal relatedness and sequence types of the *K. pneumoniae* isolates

By ERIC-PCR, 11 different clusters (A-K) were detected, each consisting of 2 to 4 K*. pneumoniae* isolates. No similarity was seen in 8 isolates from the ICU. The distribution of the isolates with their ERIC profile has been shown in Fig. 4. Two distinct STs were seen, ST5235 and ST5313. On comparison, it was found that colistin resistance (*p* = 0.01) and mortality (*p* = 0.05) were significant attributes of ST5235 that was predominantly seen in the NICU (*p* = 0.04) from the cases of neonatal sepsis (Table 1).

**Table 1:** Comparison of characteristics of the two sequence types

Characteristics	ST5235 N = 30 (%)	ST5313 N = 7 (%)	*P* value
Carbapenem resistance	23 (76.67)	3 (42.85)	0.09
Colistin resistance	22 (73.33)	3 (42.85)	0.01*
Isolates from ICU	9 (30.00)	3 (42.85)	0.51
Isolates from NICU	17 (56.66)	0 (0)	0.04*
*bla*_OXA-48_, *bla*_NDM_ combination	11 (36.66)	1 (14.20)	0.06
*bla*_SHV_, *bla*_CTXM_	17 (56.66)	2 (28.57)	0.74
Outcome (mortality)	18 (60.00)	1 (14.20)	0.05*

^*^P < 0.05

## Discussion

The study clearly demonstrated the increasing challenges in managing XDR organisms like colistin resistant CRKp and the role of the hospital environment in sustaining such organisms. The study was important in the following perspectives. Firstly, the study describes a hospital wide outbreak of colistin resistant CRKp affecting both the adult ICU and the NICU of a tertiary care hospital. Large sustained or successive outbreaks of similar isolates are on record extending over long periods [5, 13]. Gradual emergence of CRKp followed by colistin resistant *K. pneumoniae* in these sustained outbreaks has been shown in these studies. The one common thing is that all these reports have shown the occurrence of these outbreaks in coincidence with increased consumption of colistin. The emergence of colistin resistant Gram-negative bacilli per se is an obvious indication of excessive colistin use [14]. In the present study, though not within the scope, there have been enough indirect evidence of heavy colistin use in the present study setting. The monthly audit data of the adult ICU over a period of one year had revealed usage of colistin among 10–13% of admitted patients with infection per month with mean duration of administration for 16.5 ± 2.3 days. Majority of the infections were ventilator associated pneumonia due multidrug resistant *Acinetobacter baumannii*. Besides, emergence of organisms like colistin resistant *A. baumannii* [15] and carbapenem resistant *Providencia stuartii* [16] has also been reported from the same study centre, thus hinting at considerable use of colistin. However, reports of colistin resistant *K. pneumoniae* affecting the NICU are scarce in the literature. In this context, few reports of XDR *K. pneumoniae* in neonatal sepsis has been recently reported though not colistin resistant isolates [2]. Mortality in neonatal sepsis in India is relatively high at 19–38% and increased risk of mortality has been shown to be independently associated with *K. pneumoniae* infections against other microorganisms irrespective of any associated factors [17]. Owing to the ubiquitous dissemination of CRKp in the hospital environment, Indian NICU are forced to use reserve drugs like colistin as a life saving measure.

Secondly, there have been several reports of outbreaks with CRKp with majority of them being associated with KPC. However, as against countries from Europe and USA with this type of CRKp epidemiology, the predominant carbapenem resistant *Enterobacterales* (CRE) strain in Asian countries have been the various MBLs [18], among which a shift from *bla*_NDM_ to *bla*_OXA-48_ has been observed in *K. pneumoniae* in India since 2017 [19]. Additionally, a combination of *bla*_NDM_, *bla*_OXA-48_ and *bla*_CTXM-15_ has often served as a vehicle for easy dissemination of these isolates [20]. Carbapenem resistance mediated by KPCs has been depicted as potential threats due to ‘high profile hospital outbreaks and deaths’ associated with it [4]. However, treatment options in MBL and *bla*_OXA-48_ mediated resistance are very limited for infections caused by these isolates. Consequently, the impact of these isolates is reflected in the considerable mortality caused as in this study.

Thirdly, as against the common circulating clones present throughout the globe or for that matter among those previously reported from the subcontinent among the CRKp like ST11, ST14, ST43, ST231 [21], the present outbreak detected involvement of locally circulating STs. Interestingly, two simultaneously circulating STs were noted with different characteristics. While colistin resistance was significant in ST5235, all the cases of neonatal sepsis and majority of nosocomial pneumonia from the ICU were also associated with this clone which also accounted for significant mortality. We did not find any published report of previous infections caused by these two STs, though both the STs have been noted with relevance to human and hospital environment as evident in the PubMLST isolate database.

We did not find gut colonization with *K. pneumoniae* in any of the infected or non-infected neonates throughout the study period. On the contrary, skin colonization was seen with similar isolates as that of the outbreak. However, it could not be inferred whether skin colonization preceded infection. Studies have indicated 2.6% CRKp rectal colonization rates in the NICU and infection rates as high as 18% among the carriers [22]. Simultaneously, contradicting the studies on colonization, reports on pediatric population have also shown low infection rates (3.4%) among patients colonized with CRKp (29.5%) [23]. Instead, extensive environmental contamination has often been reported as the source of such outbreaks [5, 24], a fact which could have accounted for the present outbreak. This probability is also supported by the fact that extensive cleaning of the hospital environment was done during the 3 days hospital closure owing to reduced workload which could have reduced the existing hospital reservoir. Additionally, though not well identified, a major factor for transmission of these isolates throughout the hospital could be attributed to the movement of the healthcare workers based on their rotational duties. The neonate’s continuous interaction with healthcare workers during the care giving process can also intervene with the colonization of the neonates and the NICU environment [25].

It is well recognized that overcrowded healthcare facilities often promote the easy dissemination of multidrug resistant organisms (MDROs) especially in hospitals of low-middle income countries (LMICs) [26]. Limiting patient care and implementing strict IC measures throughout the hospital is often difficult. Screening cultures for early detection of colonized neonates which could have helped in timely detection of the impending problem was not effective in our situation. Transmission of MDROs through the hands of the healthcare workers, which could be controlled by effective hand-hygiene programmes often becomes a limitation due to absence of infection control nurses (ICNs) in most of the hospitals of LMICs including ours. Studies have suggested that microbial communities are quite specific in the NICU as compared to other parts of the hospital, which can get affected by various environmental stress factors [27]. These factors like antibiotic pressure, disinfection of surfaces, microflora on hands of healthcare workers affect these microbiomes by selecting the persisters in the hospital environment. Therefore, we concluded that extensive occurrence of cases in the adult ICU and environmental contamination including the labour room contributed to the near fatal outbreak in the NICU with colistin resistant CRKp of ST5235 clone. The less virulent ST5313 clone did not affect the neonates despite being detected in the NICU environment.

The study was not without limitations. We could not monitor the entire hospital in addition to the NICU in the post outbreak period. The mechanisms of colistin resistance and quantification of colistin use during this period could not be done due to limitations of resources and lack of inventory management. Despite, the study shows the challenges in controlling XDR *K. pneumoniae* and adds to the scarcely available literature of the impact of locally circulating clones causing fatal outbreaks.

## Conclusion

Large hospital outbreaks caused by colistin resistant CRKp harbouring multiple carbapenemases (*bla*_OXA-48_, *bla*_NDM_) are real challenges for containment, especially in endemic regions. Molecular epidemiology of the isolates revealed 2 circulating sequence types with ST5235 being significantly associated with colistin resistance and mortality. The global distribution and exact role of these emerging STs should be further studied. The study adds to the data on the impact of these STs in such outbreaks.

## Data Availability

All data generated or analysed during this study are included in this published article.

## Abbreviations

K. pneumoniae: *Klebsiella pneumoniae*;
AMR: Antimicrobial resistance
XDR: Extensively drug resistant
KPC: *Klebsiella pneumoniae* Carbapenemase
MBLs: Metallo Beta-lactamases
MDR: Multi drug resistant
ESBLs: Extended spectrum Beta-lactamases
NICU: Neonatal intensive care unit
XDRKp: Extensively drug resistant *Klebsiella pneumonia*
MIC: Minimum inhibitory concentration
ERIC: Enterobacterial repetitive intergenic consensus
MLST: Multilocus sequence type
ST: Sequence type
OTs: Operation theatres
SSI: Surgical site infection
MSW: Male surgery ward
IC: Infection control
CRKp: Carbapenem resistant *Klebsiella pneumonia*
CRE: Carbapenem resistant *Enterobacterales*
MDROs: Multidrug resistant organisms
LMIC: Low-middle income countries
ICNs: Infection control nurses
